# Case Report: Transient cortical blindness following coronary angiography

**DOI:** 10.12688/f1000research.50821.1

**Published:** 2021-06-03

**Authors:** Yudi Her Oktaviono, Maureen Victoria Kawilarang, Michael Kawilarang, Ruth Irena Gunadi, Petrina Theda Philothra, Makhyan Jibril Al Farabi

**Affiliations:** 1 Departement of Cardiology and Vascular Medicine, Soetomo General and Academic Hospital, Faculty of Medicine, Universitas Airlangga, Surabaya, Indonesia; 2 Department of of Physical Medicine and Rehabilitation, Soetomo General and Academic Hospital, Faculty of Medicine, Universitas Airlangga, Surabaya, Indonesia

**Keywords:** transient blindness during coronary angiography, contrast induced blindness, transient cortical blindness, contrast reaction, case report

## Abstract

Temporary blindness, also known as transient cortical blindness, is an uncommon impediment of contrast agent usage during angiography procedures. The occurrence of blindness after a cardiac catheterization procedure is rare and its pathophysiology remains largely speculative. The most probable mechanism seems to be contrast agent-related disruption of the blood–brain barrier, possibly initiated by several predisposing factors. This case reports a 52-year-old man with transient vision loss that occurred following coronary angiography. Brain magnetic resonance imaging (MRI) showed no acute pathology and his vision spontaneously returned within approximately 15 hours post-procedure without any requirement of specific therapy. Suggesting that transient cortical blindness may have occurred following coronary angiography which subsequently self-resolved.

## Introduction

Coronary angiography is a dependable diagnostic technique with low risk (below 0.5%) of significant complications (acute cardiac failure, cerebrovascular accidents), and mortality rate under 0.2%. Contrast-induced vision loss in coronary angiography is one of its rare complication. ^1^ We describe a rare incident of transitory cortical blindness following a coronary angiography procedure, followed by discussion comparing previous temporary cortical blindness as contrast-related complication in angiographic procedures.

## Case report

### Patient information

A 52-year-old male (Asian, private employee) with stable angina pectoris came to the Cardiac Catheterization Unit in July 2019 to have a diagnostic coronary angiography (DCA). He frequently experienced chest pain and fatigue during strenuous activity, with a history of hypertension, smoking and allergic reactions to amoxicillin and shrimp. There was no family history of cardiac disease and the patient had not previously undergone a coronary angiography.

### Clinical findings

Physical examinations, electrocardiography (ECG), and laboratory data were all in normal limits.

### Diagnostic assessment

**Figure 1.**
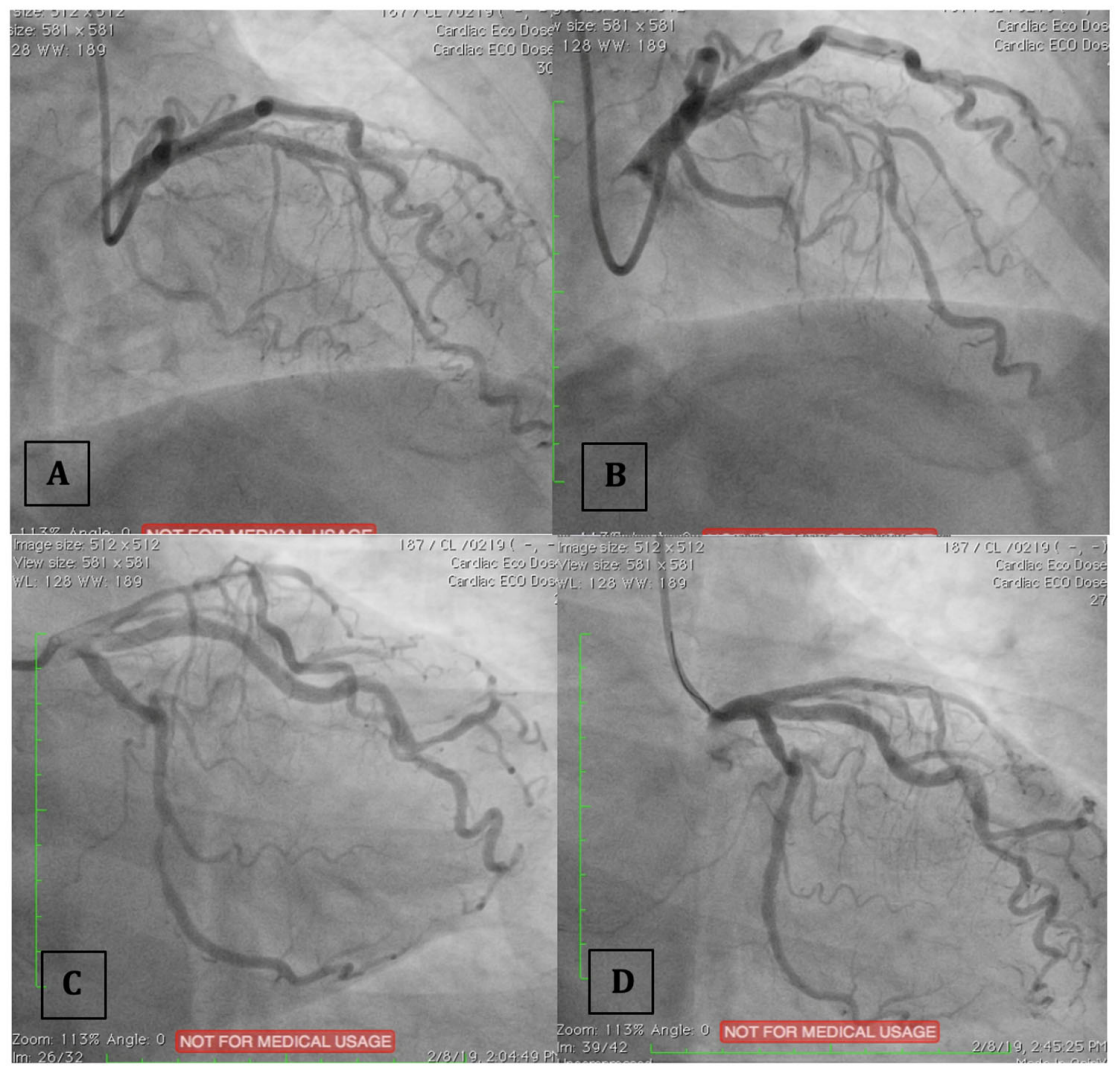
Percutaneous coronary intervention was performed at the left anterior descending (LAD) artery (A: before; B: after) and the left circumflex (LCx) artery (C: before; D: after).

The patient underwent DCA using Iopamiro contrast (non-ionic, low-osmolar iodinated contrast agent), which then revealed significant stenosis of 85% in the distal left circumflex (LCx) artery and proximal left anterior descending (LAD) branches. Percutaneous coronary intervention (PCI) was subsequently performed, however, during the stenting procedure he suddenly complained of total blindness (Visus 0/0). There were no other complaints, and vital signs was stable. Two stents were installed at LCx and LAD arteries with thrombolysis in myocardial infarction (TIMI) 3-flow results are shown in Figure 1.

**Figure 2.**
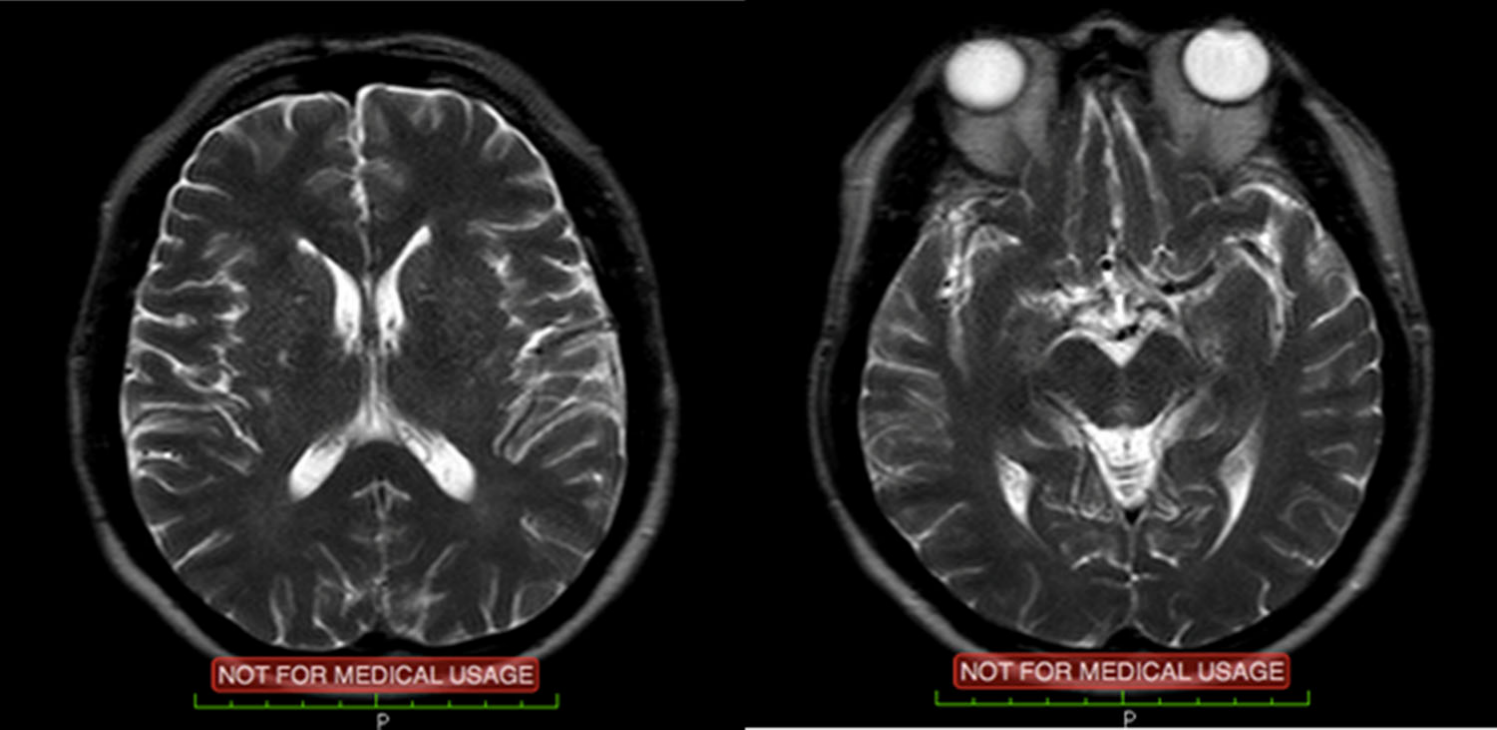
Brain magnetic resonance imaging (MRI) showing no signs of acute infarction or hemorrhagic processes.

Post PCI procedure the patient referred to the neurology department and underwent urgent non-contrast brain magnetic resonance imaging (MRI) followed by a non-contrast magnetic resonance angiography (MRA). Neither tests displayed any acute ischemic cerebral infarction ( Figure 2). Nevertheless, there were signs of chronic ischemic infarction in the left corona radiata and chronic infarction in the left paramedian cerebral pons, with multiple bilateral small vessels ischemia in the corona radiata, centrum semiovale and subcortical parietal.

### Therapeutic intervention

The patient was then given 10 mg dexamethasone intravenous (IV) bolus, maintained with 5 mg dexamethasone injection every six hours, alongside citicoline 1 gr and methylcobalamine 500mcg injection to reduce the damage that may have occurred while the blindness persisted.

### Follow up and outcome

Approximately 15 hours post PCI procedure, the patient fully regained his eyesight. There were no blurred vision or other complaints, vital signs and physical examination were within normal limits. The patient was discharged two days later with an additional diagnosis of contrast induced transient blindness.

## Discussion

Transient cortical vision loss is a complication that may occur in various types of angiographic procedures, particularly vertebral artery angiography, nevertheless this event is rare in coronary angiography. ^2, 3^ Cortical blindness is characterized by total or partial eyesight loss, while ocular movement and pupil responses are not affected. ^4^ Estimated incidence of events during/after coronary angiography is 0.05%. ^5^ The types of contrast agents (ionic and non-ionic contrast and alsoisotonic iodixanol) are associated with these complications. ^2^ The final outcome of these events are mostly satisfactory, with spontaneous gain of vision within 24-48 hours of onset without requiring any treatment.

The pathophysiology of cortical blindness itself is still unclear, various mechanism hypotheses have been propositioned such as direct toxicity from contrast agents, micro emboli, hypoxia, or cerebral edema. ^1, 3^ Immunological mechanisms are thought to be associated with the incidence of cortical blindness related to radiocontrast. ^6^ Usage of hyperosmolar solutions can increase the permeability of the blood-brain barrier in the occipital region which may facilitate contrast agents enter the visual cortex area due to poor localization during the procedure. ^7^ Patients with chronic hypertension may have higher susceptibility to increased blood-brain barrier permeability. ^8^ Once the contrast agent has been excreted, vision will return as blood-brain barrier protective function returns. ^9^ One of the main risk factors for this complication is the type of contrast agent used. The incidence of transient cortical blindness is more frequent with hyperosmolar contrast agents. Other important risk factors are uncontrolled hypertension, hypotension, kidney failure, use of immunosuppressant drugs and dehydration. ^10^ Viscosity, hyperosmolarity, and contrast agent solubility profile may contribute to increased risk of neurotoxicity, but the role of contrast volume is still debatable. ^11^ Symptoms typically begin to appear during the procedure or within the following 12 hours, with various symptoms such as cephalgia, disorientation, nausea, vomiting, decrease of consciousness, followed by sudden onset of total vision loss. ^12^

The diagnostic approach to cortical blindness involved brain imaging, which remains the gold standard to rule out other causes of acute vision loss, such as bleeding and embolic stroke, especially after intra-arterial procedures. ^13^ MRI with T2 and FLAIR is the best modality to rule out other acute causes. There are case reports of FLAIR hyper intensity in the occipital region with diffusion weighted imaging (DWI) which is nearly normal and showed a perfect resolution on repeated MRI. However, the majority of acute vision loss cases show normal MRI results. MRI is still the best diagnostic tool to detect ischemic changes in early stages, specifically for case of periprocedural cortical blindness. ^14^

Various contrast agent formulations containing iodine have been used for several years to visualize blood vessels. All of these formulations have similar iodine concentrations, but differ significantly in their structure, osmolality, and viscosity. ^15^ Types of contrast agents are divided into three generation categories, the first is high-osmolality contrast media (HOCM) generation, the second is low-osmolality contrast media (LOCM) generation, and the latest generation is isoosmolar contrast media (IOCM) where its osmolality is similar to blood. ^16^

Contrast reactions can be chemotoxic reactions or hypersensitivity reactions. Chemotoxic reactions are mostly related with the chemical nature of agent. The chemotoxic reaction also depends on the contrast dose and the speed of the contrast administration. These include volume overload, vasovagal reactions, cardiotoxicity, and nephrotoxicity. Hypersensitivity reactions, or allergic reactions, are idiosyncratic and do not depend on the rate or volume of the contrast infusion. They commonly occur immediately, within seconds to the first hour after exposure. Combination of symptoms such as pruritus, urticaria, rashes, angioedema, laryngospasm, bronchospasm, hypotension, syncope, shock, or even death may also occur. The incidence rate of mild hypersensitivity reactions is approximately 9%, and severe hypersensitivity reactions are 0.2% to 1.6%. ^17^ Death events are <1/100.000 patients. ^18^ These incidence rates are seven times higher in elderly. ^19^

The pathophysiology of hypersensitivity reactions are still poorly understood. Clinical manifestations may be similar to anaphylactic reactions; however, most hypersensitivity contrast reactions are not mediated by IgE. ^20^ The most possible etiology is an anaphylactoid reaction involving direct activation of mast cells and massive degranulation of histamine. Activation of the coagulation and kinin cascade, serotonin release, and inhibition of platelets may also play a role. ^21^ Ionic contrast such as HOCM causes more hypersensitivity reactions than non-ionic contrast LOCM. Hypersensitivity reactions occur even less frequently with non-ionic IOCM Iodixanol contrast. ^22^ An important risk factor is history of previous anaphylactic reactions to contrast media, as well as history of previous allergies or asthma. ^23^ Glucocorticoids therapy in anaphylactoid contrast reaction may improve symptoms, such as 125 mg IV methylprednisolone may be given to prevent repeated reactions in the hours following the procedure. ^15^

## Conclusion

Transient cortical blindness is a rare complication that occurs in various types of angiographic procedures, one of which is coronary angiography. The possible predisposing factors for this event include chronic hypertension and history of previous allergies. Brain MRI remains the principal diagnostic tool, with additional benefit to rule out other causes of acute vision loss, such as bleeding and embolic stroke, especially after intra-arterial procedures. Final outcome of transient cortical blindness is generally promising, with spontaneous return of vision within 24-48 hours without requiring specific therapy. Means of prevention and specific treatment are not established yet, more data and studies are required to further investigate this rare event.

## Patient perspective

From the patient’s perspective, sudden blindness after diagnostic coronary angiography was unexpected and made him afraid of permanent blindness as a consequence. The patient also considered this as a suable medical error. However, when the patient’s sight returned, the patient was extremely relieved.

## Data availability

All data underlying the results are available as part of the article and no additional source data are required.

## Consent

Written informed consent for publication of their clinical details and clinical images was obtained from the patient.
