# Supplementary material for: Case Report: Transient cortical blindness following coronary angiography
Source: F1000Res. 2022 Aug 24;10:439. Originally published 2021 Jun 3. [Version 2] doi: 10.12688/f1000research.50821.2 (PMC9433914; doi:10.12688/f1000research.50821.2)
Supplement: Peer review history

# Peer review: Reviewer response for version 2

**DOI:** 10.5256/f1000research.137751.r148426

**Published:** 2022-08-31

**Authors:** Antonia Anna Lukito

**Affiliations:** 1 Department of Cardiology and Vascular Medicine, Siloam Hospitals Lippo Village, Tangerang, Indonesia

Congratulations again to the authors.

 The authors have addressed the concerns raised in the peer review report appropriately.

 I have no further comments to make.

Are enough details provided of any physical examination and diagnostic tests, treatment given and outcomes?

Yes

Is the case presented with sufficient detail to be useful for other practitioners?

Yes

Is sufficient discussion included of the importance of the findings and their relevance to future understanding of disease processes, diagnosis or treatment?

Yes

Is the background of the case’s history and progression described in sufficient detail?

Yes

Reviewer Expertise:

I am an interventional cardiologist

I confirm that I have read this submission and believe that I have an appropriate level of expertise to confirm that it is of an acceptable scientific standard.

# Peer review: Reviewer response for version 2

**DOI:** 10.5256/f1000research.137751.r148425

**Published:** 2022-08-26

**Authors:** Zhen-Vin Lee

**Affiliations:** 1 Cardiology Unit, Department of Medicine, University Malaya Medical Centre, Kuala Lumpur, Malaysia

The appropriate revisions have been made.

Are enough details provided of any physical examination and diagnostic tests, treatment given and outcomes?

Yes

Is the case presented with sufficient detail to be useful for other practitioners?

Yes

Is sufficient discussion included of the importance of the findings and their relevance to future understanding of disease processes, diagnosis or treatment?

Yes

Is the background of the case’s history and progression described in sufficient detail?

Yes

Reviewer Expertise:

Interventional Cardiology, General Cardiology, Preventive Cardiology

I confirm that I have read this submission and believe that I have an appropriate level of expertise to confirm that it is of an acceptable scientific standard.

# Peer review: Reviewer response for version 1

**DOI:** 10.5256/f1000research.53909.r121225

**Published:** 2022-02-02

**Authors:** Zhen-Vin Lee

**Affiliations:** 1 Cardiology Unit, Department of Medicine, University Malaya Medical Centre, Kuala Lumpur, Malaysia

I first wish to congratulate the authors for successfully reporting a case that has significant educational value. I personally had a similar experience managing a patient with this rare condition in which the case had been previously published ^1^. The condition causes significant anxiety to both the treating physician and the patient. It has to be mentioned that whilst transient cortical blindness is a cause of sudden bilateral loss of vision, its incidence is extremely rare and remains a diagnosis of exclusion.

 Given that the incident happened during an invasive procedure, the possibility of other, more sinister, complications would have to be entertained and this calls for a prompt multidisciplinary involvement to ensure that the appropriate diagnosis is made and necessary therapeutic measures can be swiftly taken. 

 I agree with Dr Lukito that Figure 1A needs to be swapped with Figure 1B to correctly reflect the figure legend.

Are enough details provided of any physical examination and diagnostic tests, treatment given and outcomes?

Yes

Is the case presented with sufficient detail to be useful for other practitioners?

Yes

Is sufficient discussion included of the importance of the findings and their relevance to future understanding of disease processes, diagnosis or treatment?

Yes

Is the background of the case’s history and progression described in sufficient detail?

Yes

Reviewer Expertise:

Interventional Cardiology, General Cardiology, Preventive Cardiology

I confirm that I have read this submission and believe that I have an appropriate level of expertise to confirm that it is of an acceptable scientific standard, however I have significant reservations, as outlined above.

## References

[1] : Transient Cortical Blindness After Coronary Angiography, Bypass Graft Angiography, and Coronary Angioplasty. *Cureus* .2021; 10.7759/cureus.12542 10.7759/cureus.12542 PMC779030433425567

# Response

**Published:** 2022-02-28

**Authors:** Yudi Her Oktaviono

**Affiliations:** Department of Cardiology and Vascular Medicine, Faculty of Medicine, University of Airlangga, Soetomo Academic and General Hospital, Indonesia

Thank you very much Zhen-Vin Lee for your review and constructive feedback.

 It is indeed a rare complication which we need to be prepared.

 Yes I acknowledge that the image was swapped, I will revise it soon.

 Best Regards

 Yudi Her

# Peer review: Reviewer response for version 1

**DOI:** 10.5256/f1000research.53909.r96948

**Published:** 2021-10-20

**Authors:** Antonia Anna Lukito

**Affiliations:** 1 Department of Cardiology and Vascular Medicine, Siloam Hospitals Lippo Village, Tangerang, Indonesia

Congratulations to the author for this interesting case which also has a learning point. The writing is clear and concise. The cortical blindness during the percutaneous coronary intervention procedure is a shocking and unexpected event both for the patient and the physician. The author reported the real situation of the horrible adverse event of the percutaneous coronary intervention procedure, and provided the possible causes and the timely management, which delivered a lesson to learn for the physician. However, there is a misplacement of the procedure figures. The figures A and B, as before and after stenting figures, are apparently swapped.

Are enough details provided of any physical examination and diagnostic tests, treatment given and outcomes?

Yes

Is the case presented with sufficient detail to be useful for other practitioners?

Yes

Is sufficient discussion included of the importance of the findings and their relevance to future understanding of disease processes, diagnosis or treatment?

Yes

Is the background of the case’s history and progression described in sufficient detail?

Yes

Reviewer Expertise:

I am an interventional cardiologist

I confirm that I have read this submission and believe that I have an appropriate level of expertise to confirm that it is of an acceptable scientific standard, however I have significant reservations, as outlined above.

# Response

**Published:** 2021-11-30

**Authors:** Yudi Her Oktaviono

**Affiliations:** Department of Cardiology and Vascular Medicine, Faculty of Medicine, University of Airlangga, Soetomo Academic and General Hospital, Indonesia

Thank you very much for the feedback. It is an honor for me to be reviewed by Dr. Antonia Anna Lukito.

 We will revise the figure accordingly.

 Best Regards

 Yudi

# Response

**Published:** 2022-02-28

**Authors:** Yudi Her Oktaviono

**Affiliations:** Department of Cardiology and Vascular Medicine, Faculty of Medicine, University of Airlangga, Soetomo Academic and General Hospital, Indonesia

Thank you very much Dr Antonia Anna Lukito for all of the constructive feedback.

 Yes the figure is swapped, I will revise it accordingly.

 Regards

 Yudi Her
